# 390. HIV Mortality Trends among the United States Population from 1999-2023: A Retrospective Study using the CDC Wonder Database

**DOI:** 10.1093/ofid/ofae631.125

**Published:** 2025-01-29

**Authors:** Muhammad Sohaib Asghar, Abuoma Ekpendu, Nisar Ahmed, Muhammad Zain Khalid, Luis Duharte-Vidaurre, Chad K Brands

**Affiliations:** AdventHealth Sebring, Sebring, FL; AdventHealth Sebring, Sebring, FL; Rapido Clinica Familiar, Chicago, Illinois; University of Kentucky, Lexington, Kentucky; AdventHealth Sebring, Sebring, FL; AdventHealth Sebring, Sebring, FL

## Abstract

**Background:**

Despite the progress made in managing HIV, the mortality trends among the general population in the United States remain understudied. This lack of information hampers the ability to implement evidence-based interventions at community levels. Our aim was to analyze the trends in HIV-related mortality among US residents by demographic characteristics such as age, gender, race/ethnicity, urbanization, and US Census Regions. State and county-wide data for Age-Adjusted Mortality Rates (AAMR) were analyzed.

**Figure 1:**
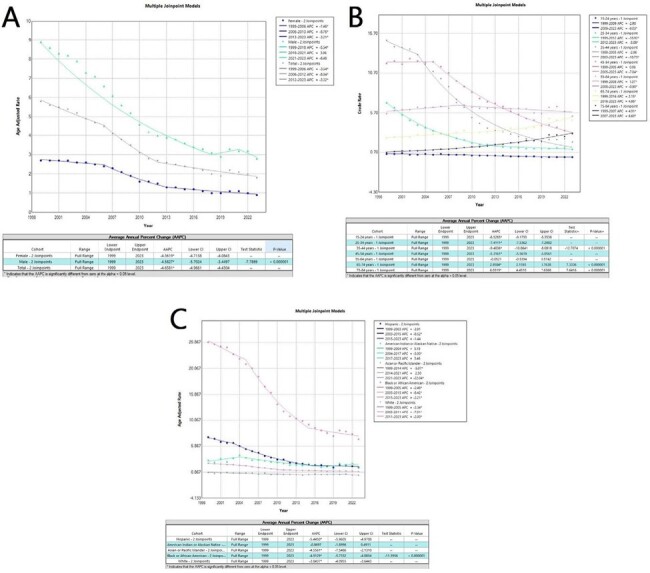
(A) AAMR of HIV reported with gender stratification, (B) crude mortality rate reported with age groups stratification, and (C) AAMR reported with race stratification. Crude mortality for females was 1.8 (AAMR=1.8), and in the male population it was about 5.3 (AAMR=5.5). For the total population, crude mortality was found to be 3.5 (AAMR=3.4). AAPC is -4.36 in females; -4.58 in males; and -4.65% overall. AAPC was increasing above 65 years of age but decreased overall below 54 years of age. AAPC is -5.44 in Hispanics, and -4.91 in Black/African Americans (with overall highest mortality). Footnotes: AAMR = Age adjusted mortality rate; AAPC: Average Annual Percent Change; and APC: Annual Percent Change.

**Methods:**

We abstracted national mortality data from the multiple cause of death files in the CDC WONDER Database. The ICD-10 codes (B20-B24) were used to identify HIV deaths from 1999-2023. Trends in age-adjusted mortality rate (AAMR) were assessed using Joinpoint regression with annual percent changes (APC). National Center for Health Statistics (NCHS) 2013 was used as Urbanization Classification Scheme for County.

**Figure 2:**
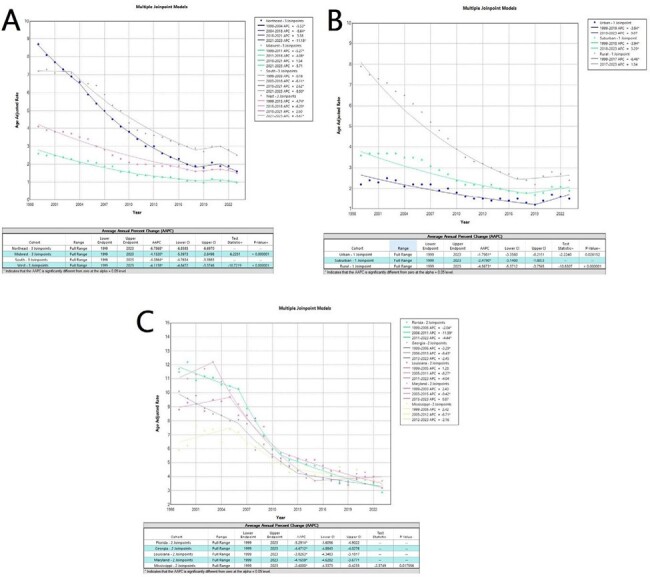
(A) AAMR reported with census-region stratification, (B) urbanization stratification, and (C) trends in top-most affected states. Crude mortality rates= Northeast: 4.5 (AAMR=4.3); Midwest: 1.7 (AAMR=1.7); South: 4.7 (AAMR=4.7); and West: 2.5 (AAMR=2.5). Crude mortality rate= Urban (large central metropolitan + large fringe metropolitan): 1.8 (AAMR=1.9); Suburban (Medium metropolitan + Small metropolitan): 2.6 (AAMR=2.6); Rural (Micropolitan + non-core = non-metropolitan): 4.4 (AAMR=4.3). States in the top 90th percentile included the District of Columbia, Florida, Maryland, Louisiana, New York, and Georgia. Footnotes: AAMR = Age adjusted mortality rate; AAPC: Average Annual Percent Change; and APC: Annual Percent Change.

**Results:**

Results were expressed as annual percentage changes (APC), average annual percentage changes (AAPC), and 95% confidence intervals (CI). Between 1999 and 2023, a total of 271,568 HIV-infected patients died within the US (AAMR=3.4 per 100,000; 95% CI: 3.3-3.5). Overall mortality trends decreased at an annual rate of -4.66% (95% CI: -4.96, -4.43) from 1999-2023 across the entire population. Specifically, the mortality trends increased among males (from the year 2018-2021), age groups 65-74 and 75-84 (overall), Non-Hispanic American Indian or Alaskan natives (from 2017-2023), across all regions (during 2018-2021), and increased slightly from 2017-2019 onwards across the urbanization divide. States in the top 90th percentile included: the District of Columbia, Florida, Maryland, Louisiana, New York, and Georgia. Union County and Miami-Dade County are highly affected within the state of Florida. Maryland showed a slight increase in trend in recent years, while Mississippi showed the slowest decline overall.

**Figure 3:**
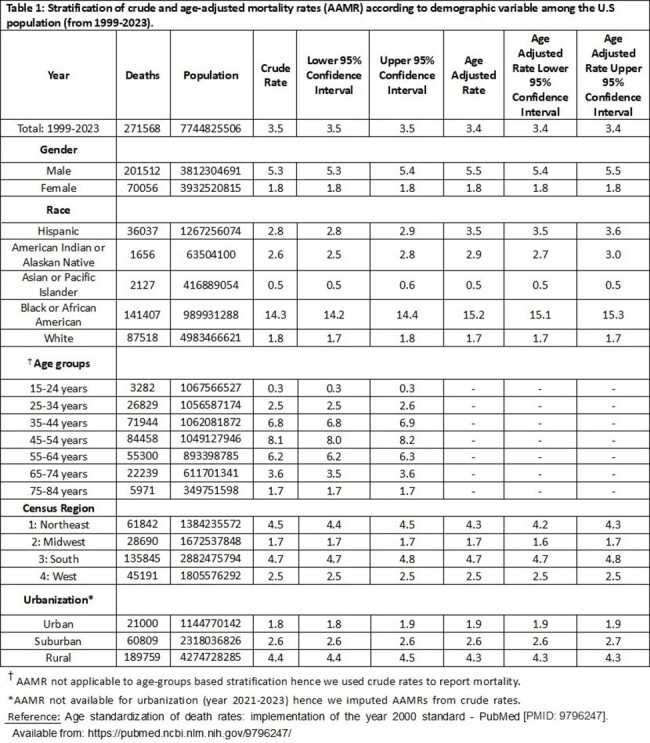
Stratification of crude and age-adjusted mortality rates (AAMR) according to demographic variable among the U.S population (from 1999-2023). † AAMR not applicable to age-groups based stratification hence we used crude rates to report mortality. *AAMR not available for urbanization (year 2021-2023) hence we imputed AAMRs from crude rates. Reference: Age standardization of death rates: implementation of the year 2000 standard - PubMed [PMID: 9796247]. Available from: https://pubmed.ncbi.nlm.nih.gov/9796247/ Footnotes: AAMR = Age adjusted mortality rate; AAPC: Average Annual Percent Change; and APC: Annual Percent Change.

**Conclusion:**

HIV mortality among the US population has decreased overall from 1999 to 2023, but with varying demographic and geographic trends. These trends highlight the need for enhanced public health surveillance to better understand the scope of HIV mortality and to identify high-risk demographic and regional subgroups for targeted interventions.

**Figure 4:**
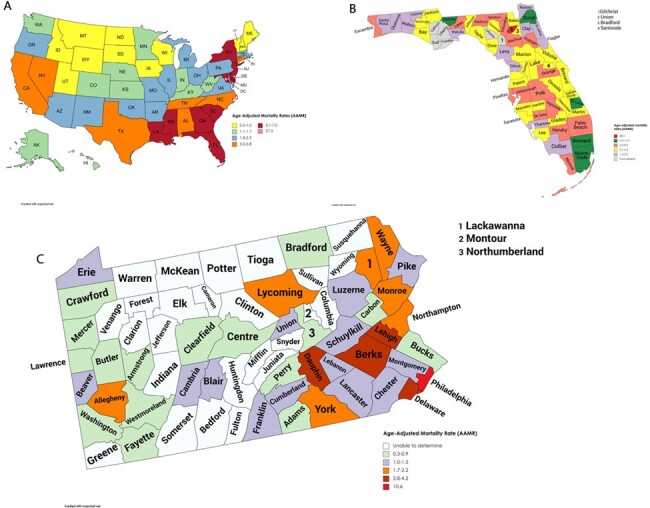
(A) State-wide heat map for AAMR reported from the year 1999-2023 for HIV mortality, (B) County-wide heat map for AAMR reported from the year 1999-2023 for the state of Florida, and County-wide heat map for AAMR reported from the year 1999-2023 for the state of Pennsylvania. Crude mortality rates= District of Columbia: 27.2 (AAMR=27.5); Florida: 7.1 (AAMR=7.0); Maryland: 7.3 (AAMR=6.9); Louisiana: 6.4 (AAMR=6.5); New York: 6.7 (AAMR=6.4); Georgia: 5.9 (AAMR=5.9); and Mississippi: 5.3 (AAMR=5.5). Union County, followed by Miami-Dade are highly affected within the Florida State which ranks second after the District of Columbia. counties which are highly affected within the State of Pennsylvania include Philadelphia, Delaware, Dauphine, Lehigh and Berks County. Footnotes: AAMR = Age adjusted mortality rate.

**Disclosures:**

**All Authors**: No reported disclosures

